# *Citrullus colocynthis* Seeds: A Potential Natural Immune Modulator Source for Broiler Reared under Chronic Heat Stress

**DOI:** 10.3390/ani11071951

**Published:** 2021-06-30

**Authors:** Mohamed I. Alzarah, Fayez Althobiati, Ahmed O. Abbas, Gamal M. K. Mehaisen, Nancy N. Kamel

**Affiliations:** 1 Department of Environmental and Natural Resources, College of Agricultural and Food Sciences, King Faisal University, P.O. Box 420, Al-Ahsa 31982, Saudi Arabia; malzarah@kfu.edu.sa; 2 Department of Biotechnology, College of Science, Taif University, P.O. Box 11099, Taif 21944, Saudi Arabia; faiz@tu.edu.sa; 3 Department of Animal and Fish Production, College of Agricultural and Food Sciences, King Faisal University, P.O. Box 420, Al-Ahsa 31982, Saudi Arabia; 4 Department of Animal Production, Faculty of Agriculture, Cairo University, Gamma St., Giza 12613, Egypt; gamoka7@gmail.com; 5 National Research Centre, Department of Animal Production, El Buhouth St., Dokki, Giza, Cairo 12622, Egypt

**Keywords:** *Citrullus colocynthis*, heat stress, broiler, humoral immunity, cell-mediated immunity, production performance, stress markers

## Abstract

**Simple Summary:**

Chronic heat-stress exposure directly affects broiler immune response. Immunosuppression was demonstrated in broiler exposed to chronic heat stress with deterioration in humoral and cell-mediated immune responses. Natural immune modulator material is considered as a safe material for human consumption of poultry products. *Citrullus colocynthis* (CC) is a natural herb plant that has traditionally been used in folk remediation. Anti-diabetic, antioxidant, and anti-inflammatory attributes are some of the CC medical properties. The present study aims to investigate the immune modulator potentials of CC seeds supplementation to chronically heat- stressed-exposed broilers. Results demonstrated that CC seeds supplementation to heat-stressed broilers was able to alleviate the negative impacts of heat stress on broiler immune responses and antioxidant status. Thus, CC seeds are suggested to be added to broilers reared under heat stress in order to improve the immune response and consequently ameliorate productivity.

**Abstract:**

There is an extensive search for natural products that can be introduced to broiler rations to improve performance, especially during the unfavorable breeding conditions. Under heat-stress conditions, the immune response seriously deteriorates, which consequently impairs broiler production performance. Thus, the present study aimed to investigate the potentials of *Citrullus colocynthis* seeds (CCs) supplementation to modulate the immune response of broilers subjected to chronic heat stress. A total of 300 Cobb-500 male broiler chickens aged 21 days were randomly divided into two equal groups and reared under either thermo-neutral condition (24 ± 1 °C) or subjected to cyclic heat stress (34 ± 1 °C for 8 h). Each group was further divided into two groups (5 replicate × 15 chicks) and was fed either the basal diet or the basal diet with 0.1% CCs supplementation. The results showed that heat stress impaired the production performance by lowering the final body weight and feed intake as well as impairing feed conversion. The levels of stress markers (i.e., malondialdehyde, tumor necrosis factor-α and corticosterone) increased (*p* < 0.05), whereas the activity of antioxidant enzymes decreased in broilers exposed to heat stress. Further, heat stress caused direct suppression of broiler humoral and cell-mediated immune responses. The stimulating index of T and B lymphocytes proliferation, as well as the antibody titer against sheep red blood cells, were significantly (*p* < 0.05) reduced by heat-stress exposure. However, CCs supplementation to broilers subjected to heat stress improved (*p* < 0.05) the final body weight, feed intake, and feed conversion ratio (FCR), compared to the non-supplemented stressed group. The cellular and cell-mediated immune response indicators significantly enhanced (*p* < 0.05) with CCs supplementation. Supplementation of CCs to broilers reared under similar environmental conditions elevated the total white blood cells (TWBCs) count and the broiler stimulating index of T and B lymphocytes. It can be concluded that CC seeds can be effectively used to stimulate the immune response and improve the production performance of broilers reared under heat-stress condition.

## 1. Introduction

In contemporary broiler industry, one of the challenging conflicts to maintain normal production performance is achieving optimum immune response. Heat-stress exposure mediates a number of physiological and behavioral changes that directly impair broiler production performance and cause a severe immunosuppression [1,2,3]. Irrespective of the broiler-strain differences, a reduction in final body weight, feed intake, daily gain, feed efficiency, and production index with high mortality rate were all associated with chronic heat-stress exposure [4,5,6,7,8]. A strong correlation was observed between the low production performance and the immunosuppression in broilers reared under either heat- or cold-stress conditions [7,9]. Decreased antibody titers against sheep red blood cells (SRBC) and Newcastle disease virus, associated with low relative weights of spleen, thymus, and bursa of Fabricius were reported in broilers subjected to chronic heat stress [5,7,9,10]. Hirakawa, et al. [11] illustrated multiple immune abnormalities, including impaired T- and B-lymphocytes proliferation and differentiation, alongside direct negative effects on primary and secondary lymphoid organs in broiler chickens subjected to chronic heat stress. Chronic heat-stress exposure activates the hypothalamic-pituitary-adrenal axis (HPA), leading to the increased production of glucocorticoid hormones that directly affect immune response causing immunosuppression [12]. Furthermore, under chronic heat stress, the abnormal immune responses were reported to be mediated by the increased production of inflammatory factors (e.g., TNF-α, HSP-70), inducing unbalance immune homeostasis in broilers [13] and rabbits [14]. A direct negative effect of heat stress on immune cell protein synthesis was found in broiler chickens [15]. Similar negative effect of heat stress on immune responses was reported in commercial laying hens [16]. Inhibition of innate immunity and increase cell death mediated by different pathway includes up-regulation of apoptotic proteins genes and increased serum pro-inflammatory cytokines levels were all presented as potential signaling pathways of heat-stress immunosuppressive effect [3]. These data suggest that, under chronic heat stress, birds are more susceptible to viral and bacterial infection, as well as low protection under different vaccination programs. Heat-stress immunosuppression effect increases the venerability of birds to exogenous microorganism infection as well as poor vaccination outcomes. Thus, in order to improve broiler performance and prosperity under high environmental temperature conditions, the immunosuppression should be alleviated to retrieve the immune homeostasis.

*Citrullus colocynthis* (CC), bitter apple fruit, is an annual plant grown in the desert. *C. colocynthis* has traditionally been known to have pharmaceutical activities. Fruit pulp, seed, leaf, and root are altogether considered to have medicinal properties [17]. The medicinal benefits of CC include antidiabetic, anticancer, antioxidant, antimicrobial, and anti-inflammation potentials [17,18,19,20]. The phytochemical studies of CC seed oil showed a reasonable quantity of phenols with anticancer activity that correlated with its fatty acids profile [21]. Ten bioactive compounds were characterized in CC seeds, including quinic acid, isovitexin, scoparin, vitexin-2’’-O-rhamnoside, reserpine, digitoxin, triprolidine, naringenin, linoleic acid, and oleic acid [22]. Anti-inflammation properties of CC seeds fractions have been reported [23,24,25] as well as anti-microbial potentials [26]. Normal growth performance was achieved in broilers fed a diet containing whole CC seeds up to 15% [27] and with no toxicity effect of their oil [28]. In a rabbit model study, CC seeds extract was reported to have immune-stimulating activity in parallel with low toxicity effect compared to the pulp extract [29]. However, to our knowledge, the employment of CC seeds in broilers diet to mediate immune response amelioration under heat-stress conditions has never been advocated. Thus, the present study was designed to investigate the potency of CC seeds supplementation to broiler chickens diet on improving production performance and alleviating immunosuppression under chronic heat-stress conditions. 

## 2. Materials and Methods

### 2.1. Ethics Compliance Statement

All experimental protocols were approved by the Research Ethics Committee (REC) at King Faisal University (KFU-REC/2021-02-17). To minimize birds suffering, heat-stress groups were monitored closely during the heat wave to detect any signs of chronic stress, such as breathing difficulty, watery discharge of the peak, decreased appetite, ruffled feathers, or droopy looking throughout the experimental period. Accordingly, if any of chronic stress sign appears, cervical dislocation was applied immediately to allow humane endpoints. 

### 2.2. Preparation of CC Seeds Powder

Dry ripe CC fruits were obtained from different herbalists in Al-Ahsa, Saudi Arabia. Fruits were sliced and the seeds were manually isolated from the pulp. Mature black seeds were selected and then pulverized in a grinder (Moulinex Type LM201, Mayenne, France). The resultant powder was collected and kept in dried containers at 4 °C until later use in the experiment.

### 2.3. Birds’ Management and Experimental Design

A total of 300 one-day-old male broiler chicks (Cobb500™) were purchased from a local hatchery and were raised in two identical environmental closed-system floor house chambers with similar conditions of size, ventilation, humidity, temperature, light intensity, and light schedule. Birds were allowed free access to feed and water that met NRC (1994) recommendations and the guidelines of Cobb500 (available at: https://www.cobb-vantress.com/enUS/products/cobb500/; accessed on 15 January 2021). A brooding temperature was maintained at 33 °C for the first 3 days of age and decreased to 30 °C for the rest of the first week. Then, the temperature was gradually reduced (3 °C per week) to reach 24 °C at 21 days of age. The lighting program was set during the first 3 days of age at 24 L: 0D, and at 23 L: 1D for the rest of the experimental period. At 21 days of age, broiler chicks were symmetrically divided into 2 × 2 factorial design, each group contained 5 replicates of 15 broiler chickens. Each replicate of birds was reared in a floor pen (1.35 m lengths × 1.35 m widths) with 5-cm deep hardwood shavings litter. Starting from 22 to 42 days of age, broilers in the first chamber were exposed to 24 ± 1 °C and 50% RH representing thermo-neutral condition (control group), while broilers in the second chamber were exposed to cyclic heat stress as a heat wave from 9:00 a.m. to 5:00 p.m. at 34 ± 1 °C temperature and 50% RH representing chronic heat stress (HS group). Under the same environmental conditions, birds in each chamber were further divided into two equal groups. One of them was given the basal diet (Table 1) and the second was given the basal diet mixed with 0.1% of *Citrullus colocynthis* seeds powder, (1 g CCs powder/kg diet). The productive performance traits were recorded for each treatment group as described below. At the end of the experimental period (42 days of age), blood samples from birds of all treatment groups were taken and prepared for further assays of some stress indicators, antioxidant markers, immunological parameters, and some biochemical measurements.

### 2.4. Production Performance Traits

Body weight was recorded at 22 and 42 days of age and the body weight gain (g/bird; BWG) was determined for each experimental group replicates considering each replicate pen as experimental unit. Feed intake (g/bird; FI) was determined for each treatment group by subtracting feed residual from the total offered amount. Then, feed conversion ratio was calculated (g feed intake/g body weight gain; FCR) for each treatment group. The body weight, total feed offered and feed residual were determinate using digital electronic balance with 0.1 g sensitivity and 20 g accuracy/200 kg weight capacity (Stip, SAMURAI, Haryana, India).

### 2.5. Blood Sampling and Preparation

At the end of the experimental period (42 days of age), ten birds from each treatment group (2 birds per replicate) were randomly selected and blood samples (≈3 mL each) were withdrawn from the brachial vein using heparinized syringes. Small drops of the whole blood were used to evaluate some hematological parameters such as total white blood cells (TWBC) count and heterophils/lymphocytes (H/L) ratio. Samples were then centrifuged for 20 min at 4 °C and 1800× *g*, and the plasma was separated and kept at −20 °C for the analysis of corticosterone, and some biochemical measurements, such as total protein (TP), albumin, globulin, aspartate aminotransferase (AST), alanine transferase (ALT), creatinine, and urea.

Another ten blood samples were obtained from each treatment group (2 samples per replicate; 4 mL each) for the isolation of peripheral blood mononuclear cells (PBMCs) according to the method previously described by Abass, et al. [30]. PBMCs were carefully isolated, washed, and suspended in culture medium, and then finally, PBMCs pellets were collected by centrifugation and stored at −70 °C for further processing. The stored PBMCs were later used for the analysis of some stress indicators, including malondialdehyde (MDA) and tumor necrosis factor-α (TNF-α), and some antioxidant markers, including total antioxidant capacity (TAC) and superoxide dismutase (SOD). In addition, heat shock protein-70 (HSP-70) expression in PBMCs was analyzed using the Western blot technique.

Further blood samples (approximately 3 mL) were collected at the end of the experimental period from ten birds in each treatment group (2 birds per replicate). PBMCs were obtained from these samples and processed freshly to measure the proliferation activity of T- and B-lymphocytes.

### 2.6. Stress Indicators and Antioxidant Markers

#### 2.6.1. H/L Ratio

H/L ratio was determined according to the methodology described by Mehaisen, et al. [31]. Blood smears (*n* = 10; 2 samples per replicate) were prepared for each treatment group, and leukocytes were stained using Hema-3 stains (22–122,911, Fisher Scientific, Pittsburg, PA, USA). The differential leukocyte counts were performed for a total of 200 leukocytes in two different slides using a light microscope (a magnification of 1000× with oil immersion) and the H/L ratio was then calculated.

#### 2.6.2. Plasma Corticosterone

Plasma corticosterone concentrations were determined (*n* = 10; 2 samples per replicate in each group) using a specific chicken corticosterone ELISA kit (MBS701668; MyBioSource, San Diego, CA, USA). The intra- and inter-assay coefficient of variations (CV) were <8% and 10%, respectively. The assay sensitivity was less than 0.0625 ng/mL, with a dynamic range of 0.5 to 20 ng/mL. The measurements were recorded by an automated microplate reader (Model 550 Microplate Reader, Bio-Rad Laboratories Inc., Hercules, CA, USA).

#### 2.6.3. MDA and TNF-α Levels in the PBMCs

The collected and stored PMBCs pellets were re-suspended in 1 mL PBS, kept on ice for 1 min, and then sonicated for another 1 min. The homogenized cells were centrifuged at 1030× *g* for 15 min at 4 °C and the supernatants were collected for the analysis. The levels of MDA and TNF-*α* in the supernatant (*n* = 10; 2 samples per replicate in each group) were quantified using ELISA kits specifically designed for chicken (MBS260816 and MBS2509660, respectively; MyBioSource, San Diego, CA, USA), and the measurements were recorded by the automated microplate reader. According to the manufacturer, the intra-assay and inter-assay CV were less than 8% and 12% for the MDA, and 5.57% and 5.89% for the TNFα, respectively. The detection ranges were 1.56–100 ng/mL and 31.25–2000 pg/mL for MDA and TNF-α, respectively.

#### 2.6.4. TAC and SOD Levels in the PBMCs

TAC and SOD were determined as antioxidant markers in the PBMCs (*n* = 10; 2 samples per replicate in each group). As mentioned above, the PBMCs pellets were homogenized, centrifuged, and the supernatant was used for the assay. The levels of TAC and SOD were determined in the supernatant following the instructions of colorimetric assay kits (MBS2540515 andMBS2563691, respectively; MyBioSource, San Diego, CA, USA), and the measurements were obtained using the automated microplate reader. The average intra- and inter-assay CV were 2.7% and 8.2% with a detection range of 0.62–145.2 U/mL for TAC, while the intra- and inter-assay CV were 5.1% and 9.6% with a detection range of 1.35–62 U/mL for SOD.

### 2.7. Expression of HSP-70 in PBMCs

The expression of HSP-70 in the PBMCs pellets (*n* = 10; 2 samples per replicate in each group) was analyzed by Western blot technique using the protocol described by Abass, Kamel, Khalifa, Gouda, El-Manylawi, Mehaisen and Mashaly [30]. Briefly, 40 μg of the total protein was loaded and separated on 12% polyacrylamide gel containing sodium dodecyl sulphate. Separated proteins were then transferred in Tris-glycine buffer containing 20% methanol, to poly-vinylidene difluoride membranes using a tank transfer for 2 h at 300 mA. Skim milk (5%) was used to block membranes for 1 h and incubated overnight at 4 °C with diluted primary anti-rabbit IgG polyclonal antibody against HSP-70 (1:1000; Cell Signaling Technology, Inc., Danvers, MA, USA). After incubation, a horse radish peroxidase conjugated secondary antibody against rabbit IgG (1:1500; Santa Cruz Biotechnology, Inc., Dallas, TX, USA) was added. Monoclonal *β*-actin antibody (1:1000; Santa Cruz Biotechnology, Inc.) was added and incubated to the membrane to confirm equal loading of samples followed by a horse radish peroxidase conjugated goat anti-mouse IgG (1:1000; Santa Cruz Biotechnology, Inc.). Detection of HSP-70 was then performed using the ECL chemiluminescence kit (GE Healthcare Life Sciences, Amersham Place, Little Chalfont, Buckinghamshire, UK).

### 2.8. Immunological Parameters

#### 2.8.1. TWBCs Count

TWBCs count (*n* = 10; 2 samples per replicate in each group) was performed according to the methods previously described by Gehad, et al. [32]. In brief, 10 μL of the whole blood was mixed with 490 μL of brilliant cresyl blue dye, and then the total leukocytes were counted under a microscope at a magnification of 200× using a hemocytometer slide.

#### 2.8.2. Toe Web Swelling

Broiler cell-mediated immune response was assessed using the magnitude of toe web swelling induced by intradermal phytohemagglutinin (PHA-P) mitogen injection as described in a previous work [30]. Briefly, ten broiler chicks (2 chicks per replicate) from each experimental group were injected with PHA-P dissolved in sterile PBS buffer (100 μg: 0.1 mL; *w*/*v*) into the toe web of the left foot between the third and the fourth digit. The swelling response to the mitogen injection was calculated as the difference in mm between the thickness of the toe web measured immediately before PHA-P injection and after 24 h of injection. 

#### 2.8.3. Antibody Titers against Sheep Red Blood Cells (Anti-SRBCs AB)

Ten broiler chicks from each experimental group (2 chicks per replicate) were assigned to evaluate the anti-SRBCs AB as a measurement of broiler humoral-mediated immune response. At 42 days of age, birds were intravenously injected with 1 mL of saline suspension of 5% SRBCs. Blood samples were collected one week later, then sera were separated by centrifugation at room temperature, 220× *g*. The anti-SRBCs AB titers were quantified by using the micro-hemagglutination technique, and the titer was expressed as log_2_ of the reciprocal of the highest dilution giving complete agglutination [33].

#### 2.8.4. Lymphocyte Proliferation Index

The stimulating index (SI) of T- and B-lymphocyte proliferation was determined according to the methods described in a previous work Abbas, et al. [34]. Briefly, viable lymphocytes (*n* = 10; 2 samples per replicate in each group) were detected from washed PBMCs using trypan blue dye (Sigma-Aldrich, St. Louis, MO, USA). The viable cells were then plated in 96-well plate at 6 × 10^6^ cells per well. To stimulate and estimate T- and B-lymphocytes proliferation, 50 mL of either concanavalin-A (a T-cells mitogen; Sigma-Aldrich, MO, USA) or lipopolysaccharide (B-cells mitogen; Sigma-Aldrich, St. Louis, MO, USA) was used; whereas, 50 mL of RPMI-1640 medium (Gibco, Thermo Fisher Scientific, MA, USA) was added to the un-stimulated control cells. Cells were then incubated for 48 h at 42 °C and 5% CO_2_. Afterwards, 15 mL of 3-(4,5-Dimethyl-2-thiazolyl)-2,5-diphenyl-2H-tetrazolium bromide (MTT) (Sigma-Aldrich, St. Louis, MO, USA) was added to each well, then cells were incubated for 4 h at 42 °C. After incubation, 100 μL of 10% sodium dodecyl sulfate, dissolved in 0.04 M HCl, was added into each well. The absorbance was detected using ELISA microplate reader at 570 nm. Stimulating indexes (SI) of T- and B-lymphocytes proliferation were then calculated as the ratio of stimulated cells optical density against the un-stimulated control cells optical density.

### 2.9. Plasma Biochemical Assay

The plasma biochemical analyses (*n* = 10; 2 samples per replicate in each group) were performed following the manufacturer instructions of available colorimetric assay kits and data were obtained using an automated microplate reader. The plasma TP, albumin (A), ALT, and AST concentrations were determined following the instructions of the kit manufacturers (ab102535, ab235628, ab241035, and ab105135, respectively; Abcam, MA, USA) as indicators for the broiler liver functions. The globulin concentration (G = TP–A) and A/G ratio were then calculated for each treatment group. Meanwhile, the plasma creatinine and urea concentrations were measured by using commercial kits (ab65340 and ab83362, respectively; Abcam, Waltham, MA, USA) to evaluate the broiler kidney function. 

### 2.10. Statistical Analysis

Data were subjected to the analysis of variance using the GLM of SAS^®^ 2004 (SAS Institute Inc., Cary, NC, USA). Heat stress, CC seeds supplementation and their interaction were set as fixed effects. Post hoc analysis was performed to compare means using Duncan’s test and the significance was set at *p* < 0.05. Results were expressed as mean ± SEM.

## 3. Results

### 3.1. Production Performance

Broiler production performance under thermo-neutral or heat-stress conditions and with or without *Citrullus colocynthis* seeds (CCs) supplementation is presented in Table 2. Heat stress negatively affected all the studied aspects of the broiler production performance. Birds exposed to heat stress showed significant reduction (*p* < 0.05) in the final body weight, body weight gain (BWG), and feed intake by 28, 41, and 24%, respectively, compared to the thermo-neutral control group. Feed conversion ratio (FCR) also showed higher values (*p* < 0.05) for broilers exposed to heat stress, reflecting poor productivity. However, CCs supplementation significantly (*p* < 0.05) enhanced FCR compared to non-supplemented broiler chickens that received basal diet. In addition, under heat-stress condition, broiler chickens supplemented with CCs showed better (*p* < 0.05) FCR compared to non-supplemented stressed group. Nevertheless, the statistical analysis did not confirm any significant differences in the other production parameters between CCs supplemented and non-supplemented groups reared under the thermo-neutral control conditions. However, under the heat-stress conditions, CCs-supplemented-stressed group was able to increase (*p* < 0.05) the final body weight and BWG by 14 and 23%, respectively, with a significant improvement in FCR in comparison with the non-supplemented stressed group.

### 3.2. Stress Indicators and Antioxidant Markers

Results showed that stress indicators (i.e., TNF-α, corticosterone, MDA and H/L ratio) were significantly increased, whereas the SOD activity and TAC were decreased when the broilers were exposed to heat-stress condition (Table 3). From one standpoint, plasma corticosterone hormone level was increased (*p* < 0.05) by 4.1-folds in the heat-stressed group compared to the thermo-neutral control group. Moreover, an exponential increase in TNF-α and MDA levels of 2- and 2.8-folds, respectively, was observed in the heat-stressed group compared to the thermo-neutral control group. Furthermore, the H/L ratio was increased by 2.4-folds in the heat-stressed group, indicating long-term stress exposure. These findings confirm the induction of oxidative stress as a consequence of heat-stress exposure. However, there was a significant (*p* < 0.05) positive effect of CCs supplementation to the heat-stressed broilers with a reduction in the stress indicators and an increase in the antioxidant markers when compared to the non-supplemented stressed group.

**Figure 1:**
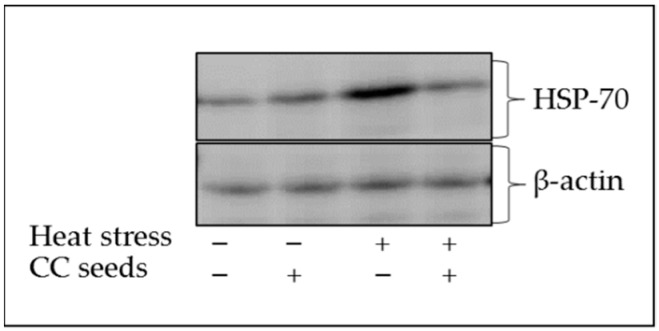
Blot of heat shock protein 70 (HSP-70) of broilers reared under thermo-neutral or cyclic heat-stress conditions and with or without *Citrullus colocynthis* (CC) seeds supplementation.

The HSP-70 Western blot output of broilers subjected to either thermo-neutral or heat-stress conditions and with or without CCs supplementation is presented in Figure 1. An over-expression of HSP-70 was noticed in the heat-stressed group compared to the thermo-neutral control group. However, CCs supplementation to heat-stressed broiler group seems to normalize the HSP-70 expression to the same levels of the thermo-neutral control group. 

### 3.3. Immunological Parameters

To assess dietary CCs immune-modulation potentials under thermo-neutral and heat-stress conditions, different humoral and cell-mediated immune assays were performed (Table 4). The results of immune response assays illustrated significant (*p* < 0.05) immunosuppression effect of heat stress on both humoral and cellular levels. The TWBCs count were deteriorated (*p* < 0.05) with heat-stress exposure. Further, a reduction (*p* < 0.05) in the humoral (i.e., Anti-SRBCs AB) as well as cell-mediated (toe web swelling) immune response was associated with heat-stress exposure. However, the supplementation of CCs to the heat-stressed broilers demonstrated positive impacts on all the examined immunological parameters. Moreover, CCs supplementation was able to bring TWBCs count as well as T- and B lymphocytes stimulating index and the antibody titer against SRBCs to the normal values of the thermo-neutral control group. Interestingly, under the same environmental conditions, CCs supplementation significantly (*p* < 0.05) increased the TWBCs count and boosted the T- and B-lymphocytes proliferation index. 

### 3.4. Plasma Biochemical Assay

Plasma biochemical parameters of broilers reared under thermo-neutral or heat-stress condition with or without CCs supplementation are presented in Table 5. Heat-stress exposure significantly (*p* < 0.05) increased the plasma total protein associated with a reduction in the albumin concentration and A/G ratio level, and an increase in the globulin concentration. Furthermore, the activity ALT and AST as well as the levels of creatinine and urea were significantly increased, indicating negative impacts of heat stress on both liver and kidney functions. Remarkably, under heat-stress conditions, CCs supplementation significantly alleviated the negative impact of heat stress on the liver enzymes activity and kidney function markers as well as the plasma albumin level. Meanwhile, the interaction effect of heat stress and CCs supplementation was significant on all the measured blood biochemical parameters except for plasma A/G ratio, plasma ALT activity, and plasma urea concentration. It is crucial to note that CCs supplementation, under thermo-neutral condition, did not show any adverse toxic effects on either broiler’s liver or kidney functions. 

## 4. Discussion

The current experiment illustrated the negative impact of heat stress on broiler production performance and immune response. The immunosuppression was confirmed with the deterioration in both cellular and humoral immune responses resultant of heat-stress exposure. The negative correlation between heat-stress exposure and immune response was demonstrated earlier [1]. One of the key factors in the immunosuppression induced by heat stress is the activation of the hypothalamic-pituitary-adrenal (HPA) axis and the elevation in corticosterone hormone secretion levels. Heat stress activates the HPA axis and initiates the production of glucocorticoid hormones, which consequently induces immune suppression [12,35,36]. Stimulation of glucocorticoid hormone release, upon stress exposure, was stated to reduce the lymphocyte proliferation and antibody production [35]. Mehaisen, Eshak, Elkaiaty, Atta, Mashaly and Abass [31] demonstrated that elevating the circulating level of corticosterone, by exogenous injection for seven successive days, was associated with poor broiler production performance and severe immune suppression (i.e., low lymphocytes stimulating index, decreased TWBC count, increased H/L ratio and a reduction in the relative weights of immune organs), and interestingly, these negative impacts were persisted even after a seven-days-recovery period. Exposure of broiler chickens to chronic heat stress decreased the proportion of T-helper (CD4+), while increased T-cytotoxic (CD8+) in peripheral blood circulation accompanied with a significant reduction in antibody responses against Newcastle and infectious-bronchitis-disease viruses [9,37]. Further, broilers reared under chronic heat-stress conditions showed a significant reduction in the antibody titer response to SRBCs with lower lymphoid organ weight compared to broilers reared under thermo-neutral conditions [38,39]. The present supplementation of CCs to heat-stressed broilers illustrated a significant elevation in the cellular and humoral immune response as well as a reduction in the stress indicators levels. These results can be partially justified by the presence of high level of tocopherols (vitamin E) in CCs oil. It is well-known that vitamin E is a potent immunomodulator [40] with anti-inflammatory properties [41]. Nehdi, et al. [42] found that CCs oil is rich in tocopherols (121.84 mg/100 g oil) with γ-tocopherol representing 95.5% of the total tocopherols content. Furthermore, Reiter, et al. [43] reviewed that γ-tocopherol has a significant anti-inflammatory and anti-oxidant activity with various molecular mechanisms. Accordingly, CCs supplementation interrupted the inflammation cascade and was able to activate immune cells proliferation. 

Heat stress is known to induce oxidative stress and excessive ROS production [44]. Broilers exposed to heat stress showed a disturbed redox status confirmed by the significant reduction in the plasma TAC accompanied by the low erythrocyte hemolysates SOD and glutathione peroxidase (GHS-Px) activities, and increased plasma MDA levels [4,45]. Using CC fruits and seeds extracts was reported to alleviate the oxidative stress by increasing SOD and GHS-Px activities, while decreasing the MDA levels in the induced-Parkinson’s-diseased mouse model [46]. The presented positive impact of CCs supplementation on redox status to heat-stressed broilers can be contributed to its antioxidant compound contents (e.g., α-tocopherol, γ-tocopherol, β-carotene, phenolic acid and flavonoids) [18,42,47,48]. 

A wide range of environmental or metabolic stress exposure induces over-expression of HSPs in order to protect cells from damage [49,50,51]. It was reported that the HSP-70 protein expression was significantly increased in different broiler breeds subjected to chronic heat stress with severe inflammation damage of the brain, muscle, and heart tissues [2]. HSP-70 was found to be associated with pro-inflammatory cytokine levels (i.e., TNF-α) in broilers subjected to chronic heat stress [49]. He, Yu, He, Hu, Xia and He [10] reported an increase in the mRNA abundances of HSP-70 in the spleen of heat-stressed yellow-feather broilers associated with increased TNF-α among other pro-inflammatory cytokines. In rat model study, it was found that CCs fractions possess anti-inflammation properties [23]. In the present study, the significant reduction in TNF-α observed with CCs supplementation can justify the associated reduction in the HSP-70 protein expression.

Blood biochemical profile reflects the physiological fitness of birds. In the present study, a significant negative effect of heat stress was observed in both liver and kidney functions. The reduction in albumin levels and the increase in creatinine, urea, and uric acid levels, as well as the increase in AST and ALT activities were associated with heat-stress exposure [4,14,45]. Liver and kidney dysfunctions were reported in rabbits subjected to heat stress and linked to the cytotoxicity induced by the increasing levels of stress hormones, inflammatory cytokines, and higher infiltration of NK and γδ T cells [14]. Moreover, albumin is considered as a negative-hepatic-acute-phase protein that decreased upon the release of pro-inflammatory cytokines during stress exposure [52]. CCs supplementation was able to relieve the negative impact of heat stress on kidney function and liver enzymes activity. CCs extract was reviewed to have hepato-protective properties with low toxicity effect [53]. Vakiloddin, et al. [54] documented a hepato-protective and antioxidant activity of the methanolic extract of CC fruits that was able to reduce ALT and AST levels in induced-hepatotoxic rats.

## 5. Conclusions

It can be inferred from the present study that heat stress induces immunosuppression by the excessive release of corticosterone and pro-inflammatory cytokines. Subsequently, the proliferation and differentiation of cellular and humoral immune cells were significantly reduced. Such unbalanced immune response directly influenced the production performance. However, CC seeds supplementation to heat-stressed broiler chickens was able to interrupt the cascade of inflammation-immunosuppression pathway. This interruption reduced the corticosterone and pro-inflammatory cytokine production, which subsequently alleviated the cellular and humoral immunosuppression. Thus, CC seeds can be supplemented to heat-stressed broilers to alleviate the negative impact of heat stress on production performance and regain immune response homeostasis. Further studies are needed to confirm the immune stimulation effect of CC seeds when applied at different levels.

## Figures and Tables

**Table 1:** Basal diet ingredients and nutrient content fed to broiler chickens from day 22 to 42 of age.

Ingredients	g/kg as Fed
Corn	626
Gluten meal	20.0
Soybean meal, 48% CP	292
Soya oil	25.0
Di-calcium phosphate	16.5
Limestone	7.00
Salt	4.50
Vitamin-Mineral Premix *	5.00
L-threonine	0.50
DL-Methionine	0.80
L-Lysine	1.70
Choline chloride	0.20
3-Phytase	0.80
Nutrient content	
Chemical analysis	
Metabolizable energy (kcal/kg)	3150
Crude protein, g/kg	202
Crude fat, g/kg	58.8
Crude fiber, g/kg	25.1
Ash, g/kg	5.63
Calculated analysis	
Calcium, g/kg	8.48
Available Phosphorus, g/kg	4.21
DL-methionine, g/kg	5.68
L-Lysine, g/kg	11.00
Sodium	1.40

* Premix provided the following vitamins and minerals per kg of diet according to the manufacturer: vitamin A, 1500 IU; vitamin D3, 200 IU; vitamin E, 10 mg; vitamin K3, 0.5 mg; thiamine, 1.8 mg; riboflavin, 3.6 mg; pantothenic acid, 10 mg; folic acid, 0.55 mg; pyridoxine, 3.5 mg; niacin, 35 mg; cobalamin, 0.01 mg; biotin, 0.15 mg; Fe, 80 mg; Cu, 8 mg; Mn, 60 mg; Zn, 40 mg; I, 0.35 mg; Se, 0.15 mg.

**Table 2:** Production performance of broilers reared under thermo-neutral (control) or cyclic heat-stress (HS) conditions and with or without *Citrullus colocynthis* (CC) seeds supplementation.

Parameter	Thermo-Neutral		Heat Stress		SEM	*p*-Value		
	Without CC	With CC	Without CC	With CC		HS	CC	HS × CC
BW, 22 d, g	754	738	743	757	29.35	NS	NS	NS
BW, 42 d, g	2432 ^a^	2488 ^a^	1740 ^c^	1983 ^b^	71.30	<0.0001	NS	NS
BWG, g	1678 ^a^	1750 ^a^	997 ^c^	1226 ^b^	75.16	<0.0001	NS	NS
FI, g	3134 ^a^	3189 ^a^	2375 ^b^	2443 ^b^	131.37	<0.0001	NS	NS
FCR	1.88 ^c^	1.82 ^c^	2.38 ^a^	2.00 ^b^	0.037	<0.0001	<0.0001	0.0005

Means in the same row with different superscripts significantly differ (*p* < 0.05). CC, *Citrullus colocynthis*; BW, body weight; BWG, body weight gain; FI, feed intake; FCR, feed conversion ratio.

**Table 3:** Stress markers and antioxidant activity indicators of broilers reared under thermo-neutral (control) or cyclic heat-stress (HS) conditions and with or without *Citrullus colocynthis* (CC) seeds supplementation.

Parameter	Thermo-Neutral		Heat Stress		SEM	*p*-Value		
	Without CC	With CC	Without CC	With CC		HS	CC	HS × CC
TNF-α, pg/mL	94.27 ^c^	83.95 ^c^	186.13 ^a^	151.63 ^b^	4.92	<0.0001	0.0002	0.0232
Corticosterone, ng/mL	2.36 ^c^	1.93 ^c^	9.62 ^a^	5.80 ^b^	0.56	<0.0001	0.0011	0.0067
MDA, µM/mL	1.08 ^c^	1.00 ^c^	4.07 ^a^	2.05 ^b^	0.23	<0.0001	0.0002	0.0004
H/L ratio	0.38 ^c^	0.35 ^c^	0.90 ^a^	0.67 ^b^	0.06	<0.0001	0.0361	NS
SOD, U/mL	5.47 ^a^	5.71 ^a^	3.36 ^c^	4.57 ^b^	0.28	<0.0001	0.0164	NS
TAC, U/mL	8.68 ^b^	9.54 ^a^	5.11 ^d^	7.10 ^c^	0.29	<0.0001	<0.0001	NS

Means in the same row with different superscripts significantly differ (*p* < 0.05). CC, *Citrullus colocynthis*; TNF-α, tumor-necrosis factor alpha; MDA, malondialdehyde; H/L, heterophils: lymphocytes ratio; SOD, superoxide dismutase; TAC, total antioxidant capacity.

**Table 4:** Immunological parameters of broilers reared under thermo-neutral (control) or cyclic heat-stress (HS) condition and with or without *Citrullus colocynthis* (CC) seeds supplementation.

Parameter	Thermo-Neutral		Heat Stress		SEM	*p*-Value		
	Without CC	With CC	Without CC	With CC		HS	CC	HS × CC
TWBC, 10^3^/mL	42.33 ^b^	56.83 ^a^	24.33 ^c^	46.50 ^b^	2.97	0.0001	<0.0001	NS
Anti-SRBCsAB, log_2_	6.50 ^ab^	7.33 ^a^	3.83 ^c^	5.33 ^b^	0.42	<0.0001	0.0113	NS
Tow web swelling, mm	0.34 ^a^	0.36 ^a^	0.18 ^c^	0.26 ^b^	0.02	<0.0001	0.005	NS
B-lymphocytes SI	2.40 ^b^	2.97 ^a^	0.69 ^c^	2.14 ^b^	0.15	<0.0001	<0.0001	0.0074
T-lymphocytes SI	4.93 ^b^	6.83 ^a^	2.12 ^c^	4.12 ^b^	0.30	<0.0001	<0.0001	NS

Means in the same row with different superscripts significantly differ (*p* < 0.05). CC, *Citrullus colocynthis*; SRBC Ab, sheep red blood cells antibody; SI, stimulating index.

**Table 5:** Blood biochemical parameters of broilers reared under thermo-neutral (control) or cyclic heat-stress (HS) condition and with or without *Citrullus colocynthis* (CC) seeds supplementation.

Parameter	Thermo-Neutral		Heat Stress		SEM	*p*-Value		
	Without CC	With CC	Without CC	With CC		HS	CC	HS × CC
TP, g/dL	3.35 ^c^	3.55 ^c^	5.74 ^a^	4.13 ^b^	0.19	<0.0001	0.0014	0.0001
Albumin, g/dL	1.78 ^ab^	1.86 ^a^	1.20 ^c^	1.61 ^b^	0.06	<0.0001	0.0007	0.0138
Globulin, g/dL	1.56 ^c^	1.68 ^c^	4.54 ^a^	2.52 ^b^	0.19	<0.0001	<0.0001	<0.0001
A/G ratio	1.16 ^a^	1.22 ^a^	0.27 ^c^	0.67 ^b^	0.11	<0.0001	0.0500	NS
AST, U/mL	84.96 ^c^	81.08 ^c^	139.37 ^a^	113.98 ^b^	3.28	<0.0001	0.0002	0.0037
ALT, U/mL	10.98 ^c^	10.45 ^c^	23.89 ^a^	18.87 ^b^	1.09	<0.0001	0.0186	NS
Creatinine, mg/dL	0.30 ^b^	0.31 ^b^	0.51 ^a^	0.37 ^b^	0.02	<0.0001	0.0161	0.0035
Urea, mg/dL	4.95 ^b^	4.41 ^b^	6.70 ^a^	5.39 ^b^	0.32	0.0004	0.0093	NS

Means in the same row with different superscripts significantly differ (*p* < 0.05). CC, *Citrullus colocynthis*; TP, total protein; A/G ratio, albumin/globulin ratio; AST, aspartate aminotransferase; ALT, alanine aminotransferase.

## References

[1] Nagai M., Iriki M., Kosaka M., Sugahara T., Schmidt K.L., Simon E. (2001). Changes in immune activities by heat stress. Thermotherapy for Neoplasia, Inflammation, and Pain.

[2] Xu Y., Lai X., Li Z., Zhang X., Luo Q. (2018). Effect of chronic heat stress on some physiological and immunological parameters in different breed of broilers. Poult. Sci..

[3] Ma D., Liu Q., Zhang M., Feng J., Li X., Zhou Y., Wang X. (2019). iTRAQ-based quantitative proteomics analysis of the spleen reveals innate immunity and cell death pathways associated with heat stress in broilers (*Gallus gallus*). J. Proteom..

[4] Hosseini-Vashan S.J., Raei-Moghadam M.S. (2019). Antioxidant and immune system status, plasma lipid, abdominal fat, and growth performance of broilers exposed to heat stress and fed diets supplemented with pomegranate pulp (*Punica granatum* L.). J. Appl. Anim. Res..

[5] He S., Yin Q., Xiong Y., Liu D., Hu H. (2020). Effects of dietary fumaric acid on the growth performance, immune response, relative weight and antioxidant status of immune organs in broilers exposed to chronic heat stress. Czech J. Anim. Sci..

[6] Awad E.A., Najaa M., Zulaikha Z.A., Zulkifli I., Soleimani A.F. (2020). Effects of heat stress on growth performance, selected physiological and immunological parameters, caecal microflora, and meat quality in two broiler strains. Asian-Australas. J. Anim. Sci..

[7] Olfati A., Mojtahedin A., Sadeghi T., Akbari M., Martínez-Pastor F. (2018). Comparison of growth performance and immune responses of broiler chicks reared under heat stress, cold stress and thermoneutral conditions. Span. J. Agric. Res..

[8] Zhang S., Ou J., Luo Z., Kim I.H. (2020). Effect of dietary β-1,3-glucan supplementation and heat stress on growth performance, nutrient digestibility, meat quality, organ weight, ileum microbiota, and immunity in broilers. Poult. Sci..

[9] Jahanian R., Rasouli E. (2015). Dietary chromium methionine supplementation could alleviate immunosuppressive effects of heat stress in broiler chicks1. J. Anim. Sci..

[10] He S., Yu Q., He Y., Hu R., Xia S., He J. (2019). Dietary resveratrol supplementation inhibits heat stress-induced high-activated innate immunity and inflammatory response in spleen of yellow-feather broilers. Poult. Sci..

[11] Hirakawa R., Nurjanah S., Furukawa K., Murai A., Kikusato M., Nochi T., Toyomizu M. (2020). Heat stress causes immune abnormalities via massive damage to effect proliferation and differentiation of lymphocytes in broiler chickens. Front. Veter. Sci..

[12] Padgett D.A., Glaser R. (2003). How stress influences the immune response. Trends Immunol..

[13] Song J.-H., Kim K.-J., Choi S.-Y., Koh E.-J., Park J., Lee B.-Y. (2019). Korean ginseng extract ameliorates abnormal immune response through the regulation of inflammatory constituents in Sprague Dawley rat subjected to environmental heat stress. J. Ginseng Res..

[14] Abdel-Latif M., Sakran T., Badawi Y.K., Abdel-Hady D.S. (2018). Influence of *Moringa oleifera* extract, vitamin C, and sodium bicarbonate on heat stress-induced HSP70 expression and cellular immune response in rabbits. Cell Stress Chaperon..

[15] Kamel N.N., Ahmed A.M.H., Mehaisen G.M.K., Mashaly M.M., Abass A.O. (2017). Depression of leukocyte protein synthesis, immune function and growth performance induced by high environmental temperature in broiler chickens. Int. J. Biometeorol..

[16] Mashaly M.M., Hendricks G.L., Kalama M.A., Gehad A.E., Abbas A.O., Patterson P.H. (2004). Effect of heat stress on production parameters and immune responses of commercial laying hens. Poult. Sci..

[17] Rahimi R., Amin G., Ardekani M.R.S. (2012). A Review on *Citrullus colocynthis* Schrad.: From Traditional Iranian Medicine to Modern Phytotherapy. J. Altern. Complement. Med..

[18] Hussain A.I., Rathore H., Sattar M.Z., Chatha S.A.S., Sarker S.D., Gilani A.H. (2014). *Citrullus colocynthis* (L.) Schrad (bitter apple fruit): A review of its phytochemistry, pharmacology, traditional uses and nutritional potential. J. Ethnopharmacol..

[19] Halla N., Boucherit K., Boucherit-Otmani Z., Touati F.Z., Rahmani N., Aid I. (2019). Ammodaucus leucotrichus and *Citrullus colocynthis* from algerian Sahara: Ethnopharmacological application, phytochemical screening, polyphenols content and antioxidant activity of hydromethanolic extracts. J. King Saud Univ. Sci..

[20] Ostovar M., Akbari A., Anbardar M.H., Iraji A., Salmanpour M., Ghoran S.H., Heydari M., Shams M. (2020). Effects of *Citrullus colocynthis* L. in a rat model of diabetic neuropathy. J. Integr. Med..

[21] Al-Hwaiti M.S., Alsbou E.M., Abu Sheikha G., Bakchiche B., Pham T.H., Thomas R.H., Bardaweel S.K. (2021). Evaluation of the anticancer activity and fatty acids composition of “Handal” ( *Citrullus colocynthis L*.) seed oil, a desert plant from south Jordan. Food Sci. Nutr..

[22] Jemai R., Drira R., Makni M., Fetoui H., Sakamoto K. (2020). Colocynth (*Citrullus colocynthis*) seed extracts attenuate adipogenesis by down-regulating PPARγ/ *SREBP*-1c and C/EBPα in 3T3-L1 cells. Food Biosci..

[23] Marzouk B., Mahjoub M.A., Bouraoui A., Fenina N., Aouni M., Marzouk Z. (2012). Anti-inflammatory and analgesic activities of a new cucurbitacin isolated from *Citrullus colocynthis* seeds. Med. Chem. Res..

[24] Marzouk B., Marzouk Z., Fenina N., Bouraoui A., Aouni M. (2011). Anti-inflammatory and analgesic activities of Tunisian *Citrullus colocynthis* Schrad. immature fruit and seed organic extracts. Eur. Rev. Med. Pharmacol. Sci..

[25] Marzouk B., Marzouk Z., Haloui E., Turki M., Bouraoui A., Aouni M., Fenina N. (2011). Anti-inflammatory evaluation of immature fruit and seed aqueous extracts from several populations of Tunisian *Citrullus colocynthis* Schrad. Afr. J. Biotechnol..

[26] Marzouk B., Marzouk Z., Mastouri M., Fenina N., Aouni M. (2011). Comparative evaluation of the antimicrobial activity of *Citrullus colocynthis* immature fruit and seed organic extracts. Afr. J. Biotechnol.

[27] Sawaya W.N., Daghir N.J., Khalil J.K. (1986). *Citrullus colocynthis* seeds as a potential source of protein for food and feed. J. Agric. Food Chem..

[28] Sawaya W.N., Daghir N.J., Khan P. (1983). Chemical characterization and edibility of the oil extracted from *Citrullus colocynthis* seeds. J. Food Sci..

[29] Shafaei H., Solaeymanirad J., Mahdavi R., Ostad Rahimi A.R., Rezazadeh H., Argani H., Rashidi M.R., Nazemieh H., Delazar A. (2007). The potentiating effects of *Citrullus colocynthis* extract on immune system. Med. J. Tabriz Univ. Med Sci..

[30] Abass O.A., Kamel N.N., Khalifa W.H., Gouda G.F., El-Manylawi M.A.F., Mehaisen G., Mashaly M.M. (2017). Propolis supplementation attenuates the negative effects of oxidative stress induced by paraquat injection on productive performance and immune function in turkey poults. Poult. Sci..

[31] Mehaisen G.M.K., Eshak M.G., Elkaiaty A.M., Atta A.-R.M.M., Mashaly M.M., Abass A.O. (2017). Comprehensive growth performance, immune function, plasma biochemistry, gene expressions and cell death morphology responses to a daily corticosterone injection course in broiler chickens. PLoS ONE.

[32] Gehad A.E., Mehaisen G.M., Abbas A.O., Mashaly M.M. (2008). The role of light program and melatonin on alleviation of inflammation induced by lipopolysaccharide injection in broiler chickens. Int. J. Poult. Sci..

[33] Loa C.C., Lin T.L., Wu C.C., Bryan T., Thacker H.L., Hooper T., Schrader D. (2001). Humoral and cellular immune responses in turkey poults infected with turkey Coronavirus. Poult. Sci..

[34] Abbas A.O., Alaqil A.A., El-Beltagi H.S., El-Atty H.K.A., Kamel N.N. (2020). Modulating laying hens productivity and immune performance in response to oxidative stress induced by *E. coli* challenge using dietary propolis supplementation. Antioxidants.

[35] Marketon J., Glaser R. (2008). Stress hormones and immune function. Cell. Immunol..

[36] Cruz-Topete D., Cidlowski J. (2015). One hormone, two actions: Anti- and pro-Inflammatory effects of glucocorticoids. Neuroimmunomodulation.

[37] Sohail M.U., Ijaz A., Yousaf M.S., Ashraf K., Zaneb H., Aleem M., Rehman H. (2010). Alleviation of cyclic heat stress in broilers by dietary supplementation of mannan-oligosaccharide and Lactobacillus-based probiotic: Dynamics of cortisol, thyroid hormones, cholesterol, C-reactive protein, and humoral immunity. Poult. Sci..

[38] Habibian M., Ghazi S., Moeini M.M., Abdolmohammadi A. (2014). Effects of dietary selenium and vitamin E on immune response and biological blood parameters of broilers reared under thermoneutral or heat stress conditions. Int. J. Biometeorol..

[39] Akhavan-Salamat H., Ghasemi H.A. (2016). Alleviation of chronic heat stress in broilers by dietary supplementation of betaine and turmeric rhizome powder: Dynamics of performance, leukocyte profile, humoral immunity, and antioxidant status. Trop. Anim. Heal. Prod..

[40] Lee G.Y., Han S.N. (2018). The role of vitamin E in immunity. Nutrients.

[41] Lewis E.D., Meydani S.N., Wu D. (2019). Regulatory role of vitamin E in the immune system and inflammation. IUBMB Life.

[42] Nehdi I.A., Sbihi H., Tan C.P., Al-Resayes S.I. (2013). Evaluation and characterisation of *Citrullus colocynthis* (L.) Schrad seed oil: Comparison with Helianthus annuus (sunflower) seed oil. Food Chem..

[43] Reiter E., Jiang Q., Christen S. (2007). Anti-inflammatory properties of α- and γ-tocopherol. Mol. Asp. Med..

[44] Wasti S., Sah N., Mishra B. (2020). Impact of heat stress on poultry health and performances, and potential mitigation strategies. Animals.

[45] Hosseini-Vashan S.J., Golian A., Yaghobfar A. (2015). Growth, immune, antioxidant, and bone responses of heat stress-exposed broilers fed diets supplemented with tomato pomace. Int. J. Biometeorol..

[46] Chen Y., Sa Y., Wang G., Pan X., Zhen Y., Cheng X., Zhang K., Fu L., Wang H., Liu B. (2019). The protective effects of *C**itrullus colocynthis* on inhibiting oxidative damage and autophagy-associated cell death in Parkinson’s disease. J. Taiwan Inst. Chem. Eng..

[47] Hussain A.I., Rathore H., Sattar M.Z., Chatha S.A.S., Ahmad F.U.D., Ahmad A., Johns E. (2013). Phenolic profile and antioxidant activity of various extracts from *Citrullus colocynthis* (L.) from the Pakistani flora. Ind. Crop. Prod..

[48] Bourhia M., Messaoudi M., Bakrim H., Mothana R.A., Sddiqui N.A., Almarfadi O.M., El Mzibri M., Gmouh S., Laglaoui A., Benbacer L. (2020). *Citrullus colocynthis* (L.) Schrad: Chemical characterization, scavenging and cytotoxic activities. Open Chem..

[49] Siddiqui S.H., Kang D., Park J., Khan M., Shim K. (2020). Chronic heat stress regulates the relation between heat shock protein and immunity in broiler small intestine. Sci. Rep..

[50] Kregel K.C. (2002). Invited Review: Heat shock proteins: Modifying factors in physiological stress responses and acquired thermotolerance. J. Appl. Physiol..

[51] Whitley D., Goldberg S.P., Jordan W.D. (1999). Heat shock proteins: A review of the molecular chaperones. J. Vasc. Surg..

[52] Gruys E., Toussaint M.J.M., Niewold T.A., Koopmans S.J. (2005). Acute phase reaction and acute phase proteins. J. Zhejiang Univ. Sci. B.

[53] Amin A., Hussain S. (2018). Hepato toxic or hepato protective: A review of hepatic effects of *Citrullus colocynthis*. J. Pharmacogn. Phytochem..

[54] Vakiloddin S., Fuloria N., Fuloria S., Dhanaraj S.A., Balaji K., Karupiah S. (2015). Evidences of hepatoprotective and antioxidant effect of *Citrullus colocynthis* fruits in paracetamol induced hepatotoxicity. Pak. J. Pharm. Sci..

